# Prolonged focal impaired consciousness seizures: a biomarker of drug resistance in patients with epilepsy

**DOI:** 10.1093/braincomms/fcag114

**Published:** 2026-04-02

**Authors:** Yue Liu, Yicong Liu, Xintong Guo, Ziheng Wei, Dingju Long, Sijing Yin, Yuting Luo, Siqing Chen, Jinming Zhang, Jingjing Chen, NingNing Wang, Heyu Zhang, Guanzhong Ni, Ziyi Chen

**Affiliations:** Department of Neurology, The First Affiliated Hospital, Sun Yat-sen University, Guangzhou, Guangdong 510080, China; Department of Neurology, The First Affiliated Hospital, Sun Yat-sen University, Guangzhou, Guangdong 510080, China; Department of Neurology, The First Affiliated Hospital, Sun Yat-sen University, Guangzhou, Guangdong 510080, China; Department of Neurology, The First Affiliated Hospital, Sun Yat-sen University, Guangzhou, Guangdong 510080, China; Department of Neurology, The First Affiliated Hospital, Sun Yat-sen University, Guangzhou, Guangdong 510080, China; Department of Neurology, The First Affiliated Hospital, Sun Yat-sen University, Guangzhou, Guangdong 510080, China; Department of Neurology, The First Affiliated Hospital, Sun Yat-sen University, Guangzhou, Guangdong 510080, China; Department of Neurology, The First Affiliated Hospital, Sun Yat-sen University, Guangzhou, Guangdong 510080, China; Department of Neurology, The First Affiliated Hospital, Sun Yat-sen University, Guangzhou, Guangdong 510080, China; Department of Neurology, The First Affiliated Hospital, Sun Yat-sen University, Guangzhou, Guangdong 510080, China; Department of Neurology, The First Affiliated Hospital, Sun Yat-sen University, Guangzhou, Guangdong 510080, China; Department of Neurology, The First Affiliated Hospital, Sun Yat-sen University, Guangzhou, Guangdong 510080, China; Department of Neurology, The First Affiliated Hospital, Sun Yat-sen University, Guangzhou, Guangdong 510080, China; Department of Neurology, The First Affiliated Hospital, Sun Yat-sen University, Guangzhou, Guangdong 510080, China

**Keywords:** seizure duration, video EEG, prolonged focal impaired consciousness seizure, drug-resistant epilepsy

## Abstract

The study aimed to explore whether Prolonged Focal Impaired Consciousness seizures (pFIC) predict drug resistance in patients with epilepsy and to identify the optimal seizure duration threshold for predicting this outcome. We registered all patients with epilepsy who were consecutively admitted to the Comprehensive Epilepsy Unit for long-term video-electroencephalogram (VEEG) monitoring at the First Affiliated Hospital of Sun Yat-sen University from January 2013 to December 2023. The database consisted of demographic information, clinical characteristics and electroencephalogram records. We evaluated the predictive performance of the recorded longest duration of Focal Impaired Consciousness seizures (FIC) through Receiver Operating Characteristic (ROC) analysis in patients with drug-resistant epilepsy (DRE). We developed and validated the optimal prognostic cut point for FIC longest duration for predicting DRE outcome using the Youden index. We compared the clinical and electroencephalographic characteristics between those with seizure duration shorter or longer than the cut point and developed a DRE prediction model. Within 18 924 patients admitted in the registration database, 490 patients who experienced FIC during VEEG monitoring were enrolled in this study, among whom 122 (24.9%) were diagnosed with DRE. The optimal cutoff point for the longest duration of FIC was 3 min (Youden index of 0.111, sensitivity of 47.5%, and specificity of 63.6%). 192 (39.2%) patients’ longest FIC duration was greater than or equal to 3 min, and 298 (60.8%) patients’ longest FIC duration for <3 min. There were more DRE patients in pFIC duration group than in shorter FIC group (21.5% versus 30.2%, *P* < 0.05). In multivariate analysis we found that pFIC, subclinical seizures, multiple seizure types, frontal seizure onset and known aetiology were associated with increased risk of DRE (*P* < 0.05). The FIC duration did not show multicollinearity (tolerance 0.988, variance inflation factor 1.012). We incorporated these indicators to multicollinearity analyses and established a DRE risk prediction model using five indicators: pFIC, frontal lobe origin, known aetiology, multiple seizure types, and subclinical seizures. The area under the ROC curve was 0.700. Our findings demonstrated that FIC duration ≥ 3 min was an independent predictor for DRE. The integrative prediction model incorporating pFIC, frontal lobe origin, known aetiology, multiple seizure types, and subclinical seizures facilitated early identification of high-risk patients.

## Teaser

Liu *et al*. report that focal impaired consciousness seizures lasting ≥3 min predict drug-resistant epilepsy (DRE). An integrative model incorporating prolonged seizure duration, frontal lobe origin, known aetiology, multiple seizure types, and subclinical seizures aids in early identification of high-risk DRE patients.

## Graphical Abstract

**Graphical Abstract:**
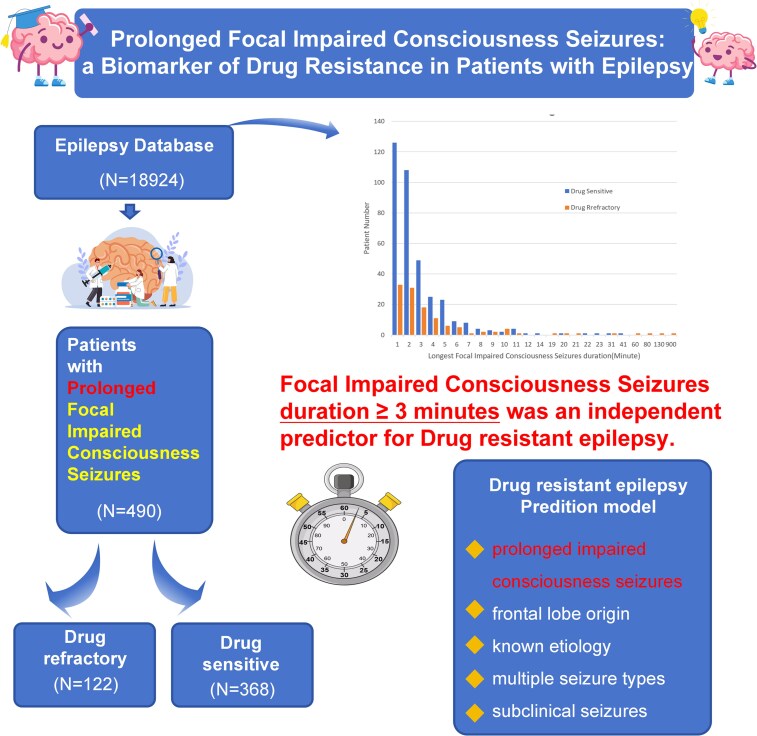

## Introduction

Epileptic seizures are transient symptoms and signs caused by abnormal and synchronized neuronal discharge activity in the brain. Currently, the international academic community has not established a unified definition for Epileptic Prolonged Seizures (EPS), which is often used interchangeably with Status Epilepticus (SE) in various clinical studies. The British National Institute for Health and Care Excellence (NICE) guidelines defined prolonged convulsive seizures as epileptic seizures lasting <5 min but >2 min longer than usual seizure duration.^1^ Recently, intranasal diazepam has demonstrated the ability to terminate seizures within 2 min.^2,3^ Some studies regarded seizures lasting >2 min without spontaneous termination as EPS.^4^ This definition has gained traction as it provided a pragmatic approach to identifying seizures that may require Rapid and Early Seizure Termination (REST).^5^

EPS represents an understudied domain and bridges the gap between SE and typical seizures. In 2015, the International League Against Epilepsy (ILAE) revised the conceptual and classification framework for status epilepticus, introducing two critical time points (T1 and T2). The revised definition emphasizes that seizure activity persisting beyond T1 may indicate failure of intrinsic seizure-terminating mechanisms or activation of pathophysiological processes leading to prolonged seizure duration. Importantly, exceeding T2 duration is associated with irreversible neuronal injury characterized by neuronal death and maladaptive reorganization of neuronal networks.^6^ SE represents a time-sensitive critical condition associated with substantial mortality.^7^ Early intervention in SE is crucial as it significantly reduces the pathological damage, increases the proportion of seizure termination, and decreases the rate of hospital readmission.^7-10^

In clinical practice, despite adequate treatment with two or more ASMs, a subset of epilepsy patients continue to experience seizures and are consequently diagnosed with drug-resistant epilepsy (DRE). The transition from an acute symptomatic event to a chronic epileptic condition is a critical juncture, as it marks the onset of the disease and is essential for identifying and studying populations with DRE. Around 6% of patients with first acute symptomatic seizure developed remote seizure^11^ while 20.4% of patients with first status epilepticus developed remote seizure, within whom up to 32% were diagnosed with DRE.^12^ Previous research has found that high-risk factors for patients with DRE also include age at onset, symptomatic epilepsy, abnormal neuroimaging findings, abnormal EEG results, psychiatric disorders, febrile convulsions, and SE.^13,14^ These patients warrant vigilant follow-up for drug resistance, with prompt referral for surgical evaluation upon confirmation of DRE. Mechanistically, EPS may impair the efficacy of benzodiazepines to terminate seizures by affecting GABA receptor function and altering the transmembrane chloride gradient, thereby affecting patient drug responsiveness.^15^

The aim of this study was to determine the optimum critical point for the longest duration of Focal Impaired Consciousness seizures (FIC)^16^ through video-electroencephalogram (VEEG) and to predict the clinical outcomes of DRE in epilepsy patients.

## Materials and methods

### Standard protocol approvals and patient eligibility

The data for this study were sourced from the epilepsy database established in 2013 at the First Affiliated Hospital of Sun Yat-sen University. We conducted a comprehensive review of medical records from consecutive patients who underwent VEEG monitoring at the Comprehensive Epilepsy Unit between January 2013 and December 2023. The patients admitted to the Epilepsy Unit for differentiation of episodes, diagnosis of new-onset epilepsy, surveillance of medication treatment effectiveness and prediction of recurrence before ASMs withdraw. The inclusion criteria for this study were epilepsy patients who exhibited clinical seizures of FIC. The exclusion criteria were: (i) patients who only experienced acute symptomatic seizures during the course of the disease, (ii) patients with severe neurological disorders other than epilepsy or serious physical conditions (such as severe hepatic, renal, cardiac, or pulmonary disfunctions), (iii) patients for whom clinical data could not be obtained, (iv) patients with a disease course <1 year and (v) patients with follow-up <1 year or three times the longest pre-treatment inter-seizure interval. The study has been approved by the Clinical Research and Experimental Animal Ethics Committee of the First Affiliated Hospital of Sun Yat-sen University (IIT-2024-899).

### Data collection

In this study, each patient underwent standard electrode placement according to the international 10–20 system, and continuous scalp electroencephalogram signals were acquired using Nicolet EEG recording equipment. We collected clinical data from these patients, including: (i) demographic characteristics: age at onset, gender, family history of epilepsy, and history of febrile seizures; (ii) EEG characteristics: duration, seizure type, frequency, EEG origin, and subclinical seizures; and (iii) prognostic information: DRE. Two experienced epileptologists combined EEG results with video recordings to strictly diagnose the seizure types according to the 2025 ILAE classification.^16^ Seizures lacking sufficient clinical data or not fitting into any defined seizure type in the 2025 ILAE classification were categorized as unknown seizure types.^16^ Seizure duration was measured from the onset timepoint, characterized by a sudden evolution in EEG frequency and amplitude, to the termination timepoint, defined as the resolution of synchronized discharges. The longest duration of epileptic seizures was recorded in minutes, rounded up to the nearest whole minute. According to the consensus definition proposed by the ILAE, Drug Resistant Epilepsy (DRE) is characterized by the inability to attain sustained seizure freedom of at least 12 months or three times the longest pre-intervention inter-seizure interval, whichever is greater, following adequate trials of two ASMs that are well-tolerated, appropriately chosen for the patients’ epilepsy type or syndrome. The interval for DRE assessment commences with the first ASM trial.^17^

### Statistical analysis

We summarized the baseline characteristics as means [standard deviation (SD)] and medians [interquartile range (IQR)] for continuous variables, and the number (percentage) for categorical variables. For group comparisons, the Mann-Whitney U-test was used for continuous variables, and the chi-square/Fisher's exact test was used for categorical variables. Receiver operating characteristic (ROC) analysis was used to assess the predictive efficacy of the longest duration of FIC and the Youden index was used to identify and validate the optimal prognostic cut point for the longest duration of FIC in predicting DRE outcomes. A *P*-value < 0.05 was considered statistically significant. Variables with *P* < 0.1 in univariate analysis were included in the multivariate logistic regression model. Multicollinearity was assessed using the variance inflation factor (VIF). Significant multicollinearity was ruled out if VIF < 1.5 or the condition number < 15. Based on significant factors identified in the univariate analysis, a predictive model for DRE was constructed.

## Results

**Figure 1:**
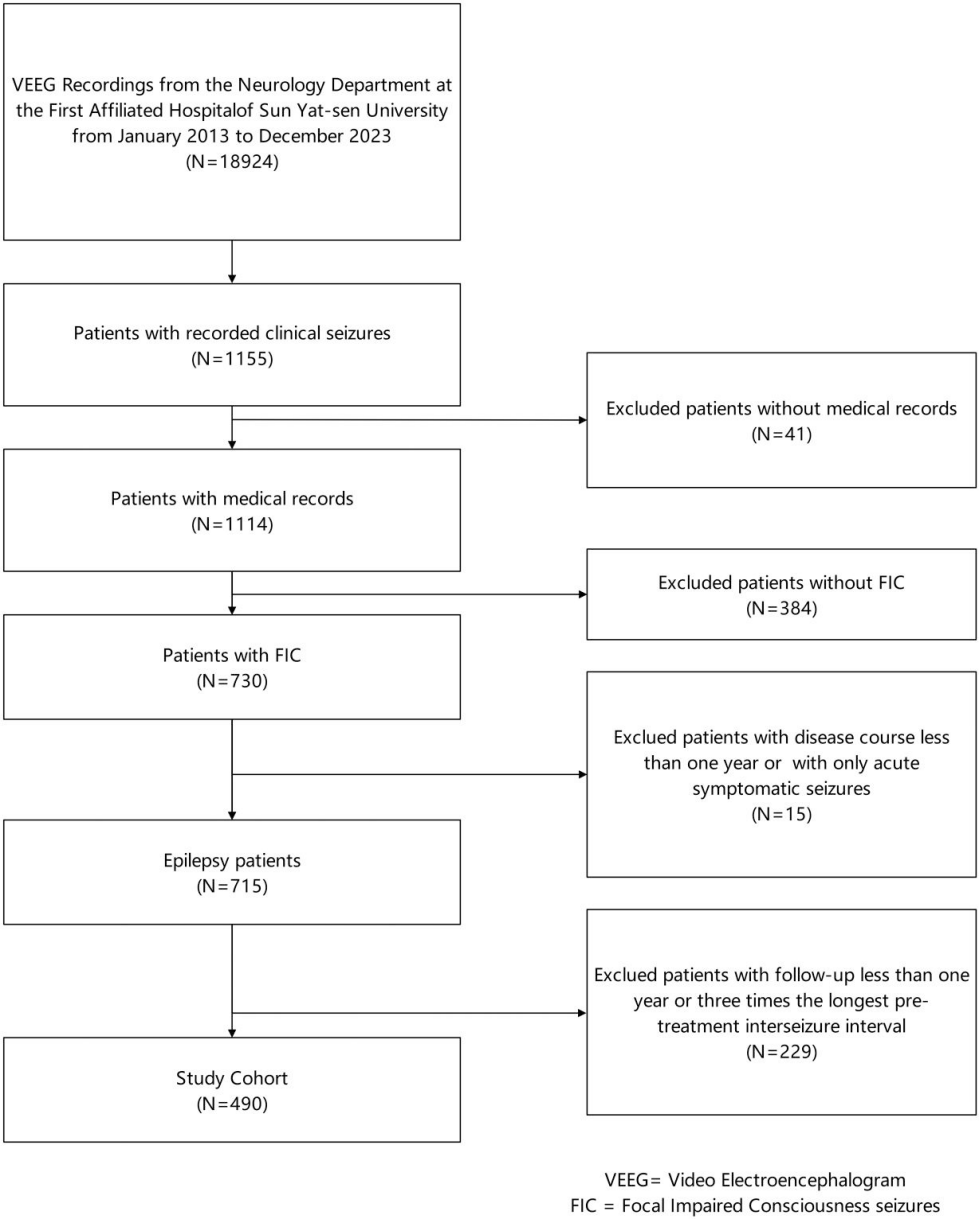
Flow-chart of patients enrolled in the study.

**Figure 2:**
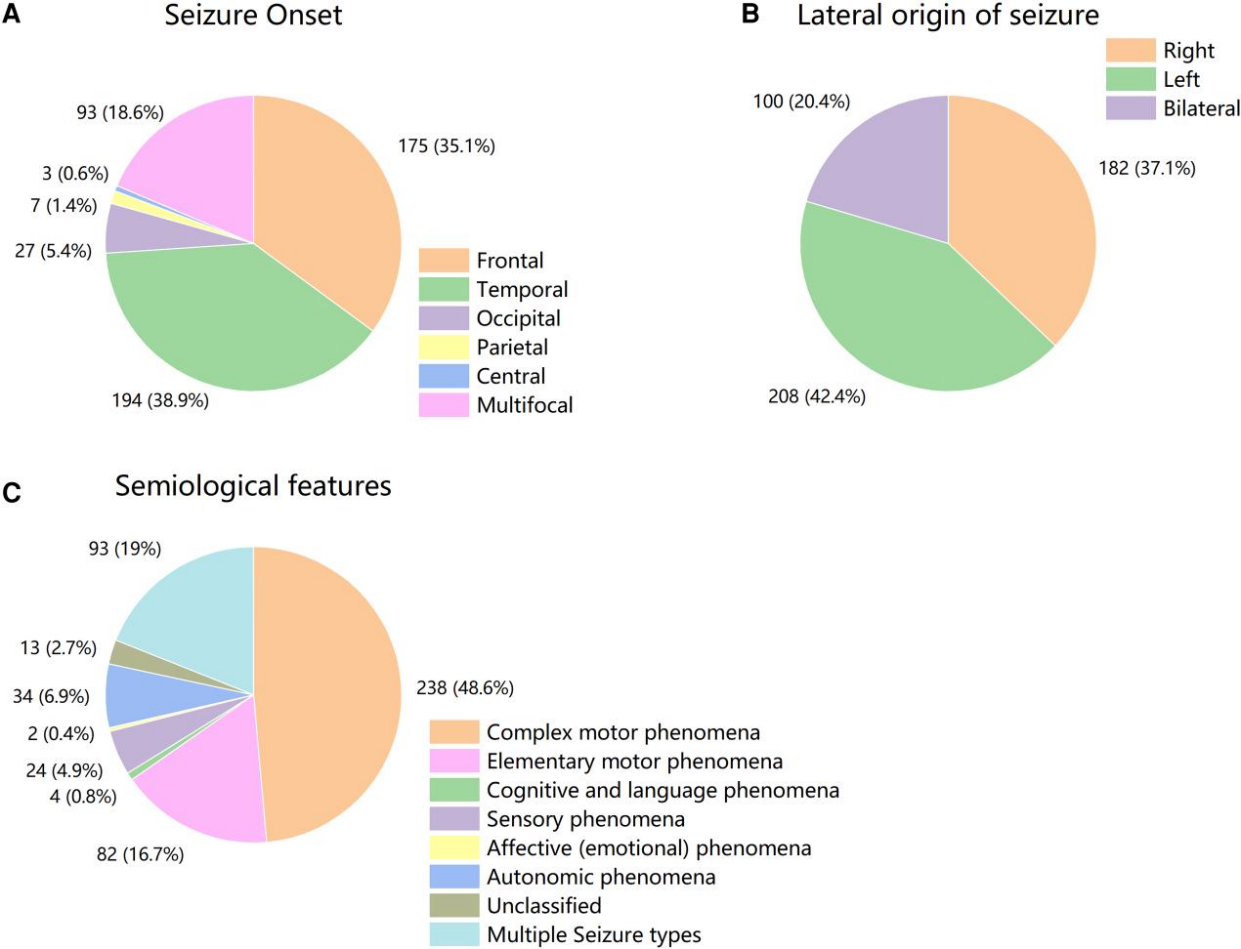
**Pie chart of seizure onset, lateral origin and seizure manifestations.** (**A**) The distribution of the seizure onset locations. The frontal lobe accounts for 35.1% (175 cases), the temporal lobe for 38.9% (194 cases), the occipital lobe for 5.4% (27 cases), the parietal lobe for 1.4% (7 cases), the central lobe for 0.6% (3 cases), and multifocal onset for 18.6% (93 cases). (**B**) The distribution of the lateral origin of seizures. Seizures originate from the right side in 37.1% (182 cases), the left side in 42.4% (208 cases), and bilaterally in 20.4% (100 cases). (**C**) The distribution of semiological features of seizures. Complex motor phenomena are observed in 48.6% (238 cases), elementary motor phenomena in 16.7% (82 cases), cognitive and language phenomena in 19% (93 cases), sensory phenomena in 6.9% (34 cases), affective phenomena in 0.4% (2 cases), autonomic phenomena in 4.9% (24 cases), unclassified features in 2.7% (13 cases), and multiple seizure types in 0.8% (4 cases).

Between January 2013 and December 2023, a total of 18 924 VEEG recordings were obtained from The First Affiliated Hospital of Sun Yat-sen University. After a detailed review and analysis of EEG data, a final cohort of 490 patients was enrolled in this study (Fig. 1). Among the 490 patients enrolled in the study, the median age at onset was 14 years (IQR: 7–26 years). The gender distribution was relatively balanced, with females accounting for 42.0% of the cohort. The median duration of the longest FIC was 2 min (IQR: 1–4 min), and 26 patients (5.3%) underwent focal SE with impaired consciousness. 36 patients (7.3%) had a history of febrile convulsions, and 122 patients (24.9%) were diagnosed with DRE (Table 1). Specific details regarding the seizure origins and seizure manifestations were illustrated in Fig. 2. Approximately one-third of the patients exhibited EEG onset in the frontal region (35.1%) and temporal region (38.9%). Approximately one-fifth of the cases originated in bilateral hemispheres (20.4%). According to the 2025 ILAE classification, complex motor phenomena were observed in approximately half of the patients (48.6%).

**Table 1:** Baseline characteristics of the patients included in the study (n = 490)

Variables	
Total, *n*	490
Age at first seizure onset (Year), Median (IQR)	14 (7, 26)
Gender, female, *n* (%)	206 (42.0%)
History of febrile convulsions, *n* (%)	36 (7.3%)
Family history of epilepsy, *n* (%)	32 (6.5%)
Longest FIC duration (Minute), Median (IQR)	2 (1.4)
Focal SE with impaired consciousness, *n* (%)	26 (5.3%)
Multiple seizure types, *n* (%)	63 (12.9%)
Presence of subclinical seizures, *n* (%)	64 (13.1%)
Known aetiology of epilepsy, *n* (%)	267 (54.5%)
Drug-resistant epilepsy, *n* (%)	122 (24.9%)

Abbreviations: DRE, Drug-resistant epilepsy; FIC, Focal impaired consciousness seizures.

**Figure 3:**
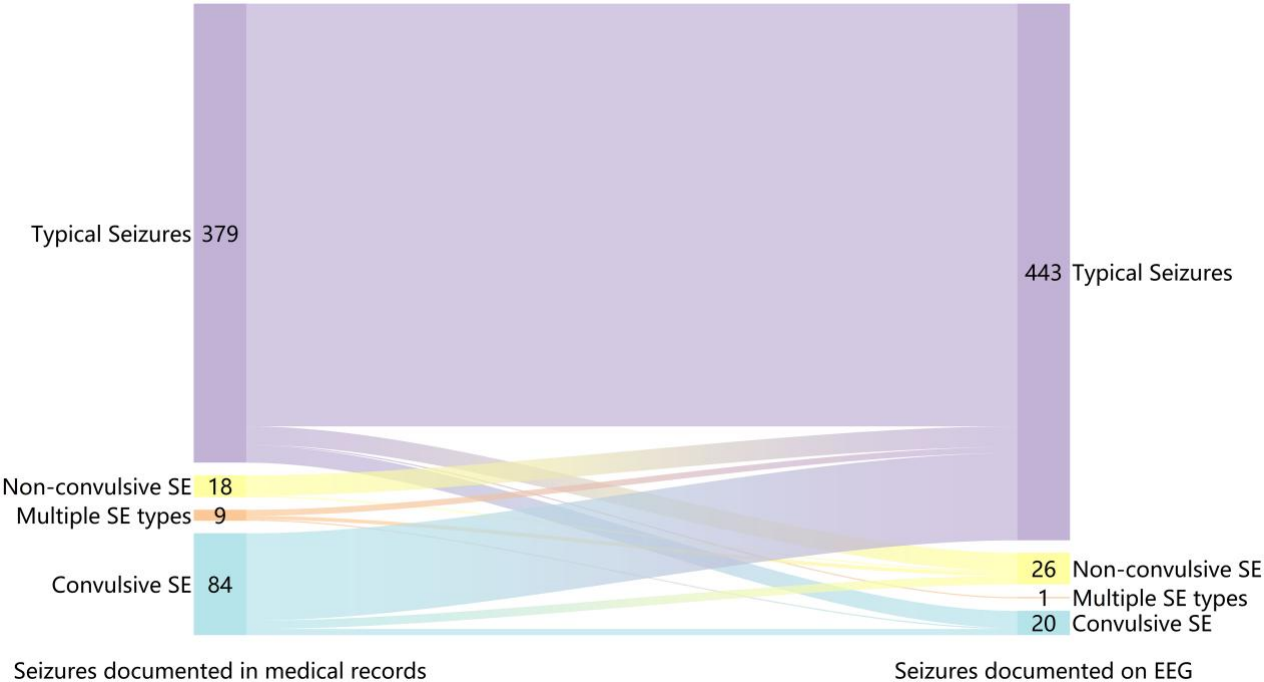
**Alluvial diagram illustrating the change in seizures by medical records or VEEG.** Medical records documented convulsive SE in 84 patients (17.1%), whereas EEG-confirmed cases only 20 (4.08%). The distribution of SE types differed significantly between the two recording methods (χ^2^ = 52.222, df = 3, *P* < 0.001). Pearson's chi-square test showed that all cells had expected frequencies ≥5 (minimum expected frequency = 5.00), meeting the test assumptions. Fisher's exact test yielded consistent results (*P* < 0.001).

**Figure 4:**
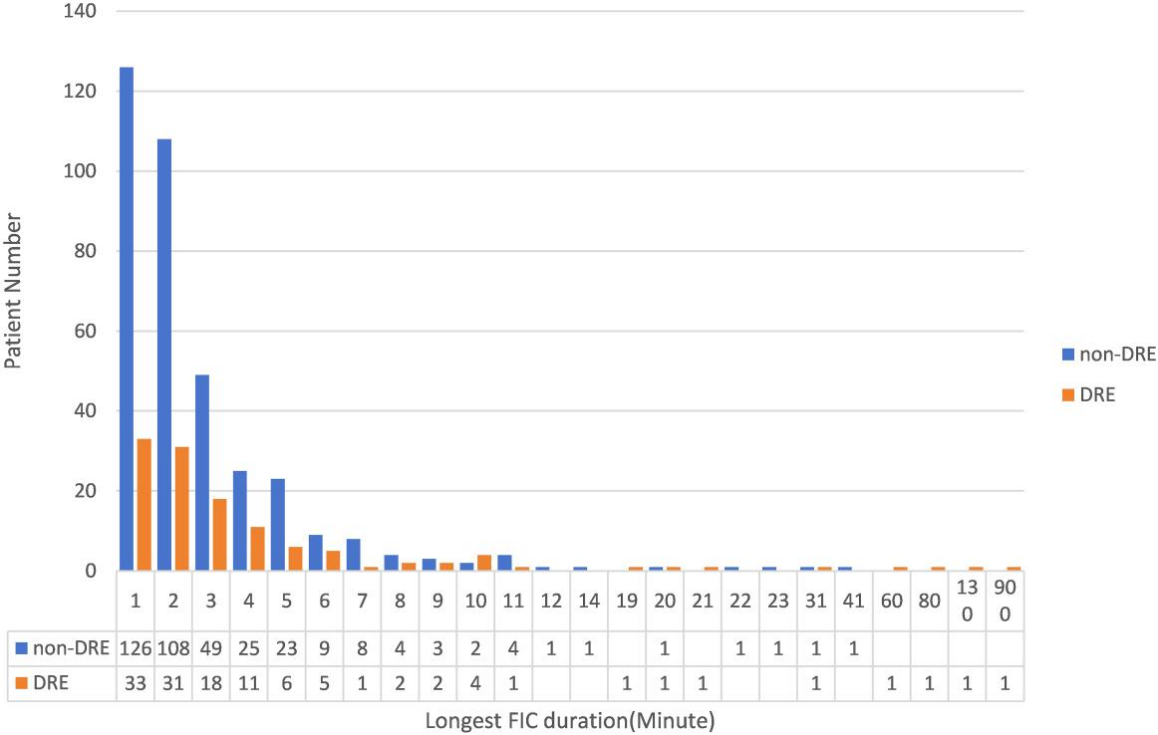
**Column chart of the longest FIC duration.** More than three-quarters of the patients had a duration within 3 min. A significant increasing trend in the proportion of patients with DRE was observed with longer seizure durations (*P* = 0.013).

To further investigate the discrepancies in different assessment methods, we conducted statistical analyses comparing data from EEG monitoring with medical records. The Results were visualized using an alluvial diagram (Fig. 3). Medical records documented convulsive SE in 84 patients (17.1%), whereas EEG-confirmed cases only 20 (4.08%). Most patients clinically diagnosed with convulsive SE exhibited typical seizures during VEEG monitoring. No statistically significant difference was observed in non-convulsive SE between two groups (3.5% versus 5.1%, *P* = 0.962). The distribution of the longest duration of FIC is shown in Fig. 4.

The results of the ROC curve and the area under the curve (AUC) with a 95% confidence interval (CI) indicated that the predictive ability of the longest duration of FIC for DRE was good [AUC 0.571, (0.511–0.631), *P* < 0.05]. The optimal cutoff point for the longest duration of FIC was 3 min (Youden index of 0.111, sensitivity of 47.5%, and specificity of 63.6%).

In this study, patients were divided into two groups based on whether the duration of FIC exceeded 3 min: the shorter FIC group and the prolonged FIC group(pFIC), regardless of whether the seizure met the criteria for SE. 192 patients’ longest FIC duration was greater than or equal to 3 min, and 298 patients’ longest FIC duration for <3 min. Table 2 compares the clinical and EEG characteristics of the two groups. The results showed that the proportion of paediatric-onset patients was very similar between the pFIC and shorter FIC groups (60.4% versus 59.1%). A higher proportion of EEG findings indicated frontal lobe origin (38.6% versus 31.3%, *P* = 0.098) in patients with shorter duration. In patients with pFIC, multifocal origins were more frequently observed (13.8% versus 27.1%, *P* = 0.000). A history of febrile convulsions (4.4% versus 12.0%, *P* = 0.002) and known aetiology (51.3% versus 59.4%, *P* = 0.081) were risk factors for patients with pFIC. The proportion of patients with DRE showed a statistically significant difference between the two groups (21.5% versus 30.2%, *P* < 0.05).

**Table 2:** The demographic and clinical characteristics of patients with different longest FIC duration

Variables	Shorter FIC *n* = 298	pFIC *n* = 192	*P* values
Age at first seizure onset, *n* (%)			
Paediatric	176 (59.1)	116 (60.4)	0.765
Gender, female, *n* (%)	128 (43.0)	78 (40.6)	0.610
History of febrile convulsions, *n* (%)	13 (4.4)	23 (12.0)	0.002*
Family history of epilepsy, *n* (%)	16 (5.4)	16 (8.3)	0.195
Multiple seizure types, *n* (%)	39 (13.1)	24 (12.5)	0.850
Seizure onset, *n* (%)			
Frontal	115 (38.6)	60 (31.3)	0.098*
Temporal	124 (41.6)	70 (36.5)	0.255
Occipital	11 (3.7)	7 (3.6)	0.979
Parietal	5 (1.7)	2 (1.0)	0.562
Central	2 (0.7)	1 (0.5)	0.835
Multifocal	41 (13.8)	52 (27.1)	0.000*
Lateral origin of seizure, *n* (%)			
Right	101 (33.9)	81 (42.2)	0.064*
Left	134 (45.0)	74 (38.5)	0.160
Bilateral	63 (21.1)	37 (19.3)	0.616
Presence of subclinical seizures, *n* (%)	37 (12.4)	28 (14.6)	0.490
Aetiology, *n* (%)			
Known aetiology of epilepsy	153 (51.3)	114 (59.4)	0.081*
Genetic aetiologies	9 (3.0)	9 (4.7)	0.338
Structural aetiologies	124 (41.6)	81 (42.2)	0.899
Epilepsy syndromes, *n* (%)			
Lennox-Gastaut syndrome	6 (2.0)	3 (1.6)	0.717
DRE, *n* (%)	64 (21.5)	58 (30.2)	0.029*

Abbreviations: DRE, Drug-resistant epilepsy; FIC, Focal impaired consciousness seizures.

We compared the clinical and electroencephalographic characteristics of patients with a DRE outcome with those who did not have DRE or had not reached the follow-up endpoint (Table 3). We found that the proportion of paediatric-onset patients in the DRE group was higher than that in the non-DRE group (66.4% versus 57.3%). Patients with pFIC were more likely to be diagnosed with DRE (34.7% versus 46.7%, *P* = 0.002). Similar to the findings of the pFIC group, the DRE group was also more likely to have known aetiology (49.5% versus 69.7%, *P* < 0.05). However, the DRE group had more patients with frontal lobe origins (33.4% versus 42.6%, *P* = 0.066), multiple seizure types (9.5% versus 23.0%, *P* < 0.05), and were more likely to have subclinical seizures (10.1% versus 23.0%, *P* < 0.05). Lennox-Gastaut syndrome was significantly associated with DRE (*P* = 0.032).

**Table 3:** The demographic and clinical characteristics of patients with DRE and non-DRE

Variables	Non-DRE *n* = 368	DRE *n* = 122	*p* values
Age at first seizure onset, *n* (%)			
Paediatric	211 (57.3)	81 (66.4)	0.077
Gender, female, *n* (%)	51 (41.0)	55 (45.1)	0.432
History of febrile convulsions, *n* (%)	24 (6.5)	12 (9.8)	0.224
Family history of epilepsy, *n* (%)	23 (6.3)	9 (7.4)	0.662
Multiple seizure types, *n* (%)	35 (9.5)	28 (23.0)	0.000*
pFIC, *n* (%)	77 (34.7)	56 (46.7)	0.002*
Seizure onset, *n* (%)			
Frontal	123 (33.4)	70 (42.6)	0.066*
Temporal	153 (41.6)	41 (33.6)	0.119
Occipital	13 (3.5)	5 (4.1)	0.773
Parietal	6 (1.6)	1 (0.8)	0.513
Central	1 (0.3)	2 (1.6)	0.093*
Multifocal	72 (19.6)	21 (17.2)	0.566
Lateral origin of seizure, *n* (%)			
Right	132 (35.9)	72 (41.0)	0.311
Left	160 (43.5)	48 (39.3)	0.423
Bilateral	76 (20.7)	98 (19.7)	0.816
Presence of subclinical seizures, *n* (%)	37 (10.1)	94 (23.0)	0.000*
Aetiology, *n* (%)			
Known aetiology of epilepsy	182 (49.5)	85 (69.7)	0.000*
Genetic aetiologies	16 (4.3)	2 (1.6)	0.168
Structural aetiologies	135 (36.7)	70 (57.4)	0.000*
Epilepsy syndromes, *n* (%)			
Lennox-Gastaut syndrome	4 (1.1)	5 (4.1)	0.032

Abbreviations: DRE, Drug-resistant epilepsy; pFIC, Prolonged focal impaired consciousness seizures.

We incorporated variables with *P* < 0.1 in a multicollinearity analysis. We found that the tolerance for pFIC was 0.988, and the VIF was 1.012, which is well below 10. This indicated that the pFIC showed no multicollinearity, and it could be assessed independently for its impact on DRE without being influenced by other independent variables.

We performed a multivariate analysis on the seven factors with *P*-values less than 0.1 from the univariate analysis results and included them in a multiple logistic regression analysis. We selected five factors to construct a risk prediction model. The formula for the model construction is: Logit (*P*) = −2.425 + 0.467 × (pFIC) + 0.856 × (Subclinical seizures) + 1.017 × (Multiple seizure types) + 0.632 × (Frontal lobe origin) + 0.981 × (Known aetiology). The Hosmer-Lemeshow goodness-of-fit test (χ^2^ = 3.38, *P* = 0.848) indicated that the model has good calibration ability. The area under the ROC curve was 0.700 (95% CI: 0.645–0.756), indicating that the model had good discriminative power. The ROC curve of the prediction model is displayed. Table 4 presents the DRE prediction risk score. A total score of 0–2 points indicates a DRE risk <25%, 3–4 points corresponds to a risk of 10–50%, 5–6 points signifies a risk of 50–75%, and 7–8 points represents a risk >75%.

**Table 4:** DRE risk prediction score

Variables	Scores
pFIC	1
Presence of subclinical seizures	2
Multiple seizure type	2
Frontal seizure onset	1
Known aetiology of epilepsy	2

Abbreviation: pFIC, Prolonged focal impaired consciousness seizures.

## Discussion

In this retrospective study, we provided detailed clinical information on 490 patients presenting with FIC at our centre and analyzed the clinical and electroencephalographic characteristics of these patients. The longest duration of FIC ≥3 min serves as the optimal predictor for DRE. Additionally, pFIC, frontal lobe onset seizures, known aetiology, multiple seizure types, and subclinical seizures are risk factors for DRE.

Compared with convulsive seizures, FIC, also known as ‘complex partial seizures’, often manifest as impairment of awareness and responsiveness, which are difficult to identify in clinical practice.^16^ Previous study has collected 375 FIC episodes from 85 epileptic patients, with most seizures lasting ≤7 min.^18^ Similarly, in our study, the median maximum duration of FIC was 2 min (IQR:1–4 min), and 92.4% of patients had seizures terminating within 7 min, which is consistent with literature reports.

The incidence of DRE varies across studies and populations, but approximately one-third of epilepsy patients were diagnosed with DRE and require surgical or alternative therapies.^13,14^ It is reported at 27% in idiopathic generalized epilepsy,^19^ 10–30% in autoimmune encephalitis,^20,21^ and 0–33% in infectious encephalitis, depending on the specific pathogen.^22^ Additionally, SE was associated with a DRE incidence of 24.2%.^23^ However, our study observed a comparatively lower DRE incidence of 24.9%. This discrepancy may stem from: (i) Restricted inclusion criteria: Only patients with VEEG FIC were analyzed; (ii) Patients who were still undergoing medication adjustment during follow-up were classified into the non-DRE group. Although these ‘gray-zone cases’ did not meet the diagnostic criteria currently, they may progress to DRE as the disease advances, potentially increasing the false-positive rate in the non-DRE group.

A retrospective study conducted in 2024 found that patients with super-refractory status epilepticus were at higher risk of DRE.^12^ Longer SE duration is independent predictors of DRE after convulsive SE.^23^ However, whether non-convulsive SE is related to DRE remains controversial.^12,23^ Data from animal models of SE provide a mechanistic basis for this link, showing that excessively prolonged epileptic activity may be associated with the formation of epileptogenic networks.^24,25^ In this context, we systematically analyzed the impact of seizure duration, specifically defining and examining EPS on the risk of DRE. The fact that EPS emerged as a statistically significant predictor in both univariate and multivariate analyses suggests that the duration of acute seizure activity is a critical determinant of long-term therapeutic outcomes. We hypothesize that EPS may not only signify acute treatment resistance but also mark an early stage in the transition towards chronic network-level changes that underlie DRE.

Additionally, focal epilepsy was significantly associated with the development of DRE (92.0%, *P* = 0.04).^26^ Notably, although this study cohort comprised both focal (75.8%) and generalized seizure types (24.2%), the vast majority of DRE cases were observed in patients with focal epilepsy. The 2019 PRO-LONG study followed epilepsy patients for at least 10 years, found that generalized epilepsy was associated with early remission (OR = 3.40) and relapsing-remitting (OR = 3.34).^27^ In paediatric DRE patients, the diagnostic yield of genetic aetiologies identified by WES can be as high as 55.5%.^28^ Approximately 20% of post-stroke epilepsy patients might be diagnosed with DRE, and the progression is closely related to factors such as younger age at onset, seizure type and severity of stroke, presence of SE, and latency of post-stroke seizures.^29-31^ Our study included epilepsy patients of all age groups, genetic and structural aetiologies were combined into the category of known aetiologies, which were also found to have a significant association with DRE.

A retrospective cohort study in 2017 found DRE to be independently associated with subclinical seizures,^32^ and similar results were observed in our study. The essence of subclinical seizures is electroencephalographic abnormalities without any accompanying clinical symptoms. This phenomenon suggests that persistent electrophysiological abnormalities, whether epileptiform discharges or subclinical seizures, may lead to changes in drug responsiveness and thus timely intervention may be required for termination.

In our study, one-third of patients had seizures originating from the frontal lobe and temporal lobe respectively, and frontal lobe onset seizures were independent predictors of DRE. Frontal lobe epilepsy is characterized by brief, frequent, and prominent motor symptoms,^33^ which aligns with our observation. However, temporal lobe epilepsy is widely regarded as the most common and representative type leading to DRE. In an Indian cohort of 150 patients with DRE, temporal lobe lesions accounted for 49.3% of all DRE cases on MRI.^34^ This inconsistency may arise from the following factors. First, differences in the study population resulted in a relatively higher proportion in the non-DRE group, potentially affecting statistical power. Second, international studies on temporal lobe epilepsy and DRE have primarily relied on surgical case cohorts,^35^ lacking control groups and systematic analysis of the correlation between the origins of different brain regions and their association with DRE risk. Third, temporal lobe epilepsy is often associated with hippocampal sclerosis, the incidence of drug-resistant mesial temporal lobe epilepsy with hippocampal sclerosis is estimated at 2.3–4.3 cases per 100 000 people per year.^36,37^ We hypothesize that the structural aetiology of temporal lobe epilepsy, rather than its origin in specific brain regions, represents a key influencing factor for DRE. Meanwhile, our cohort may have included a relatively high proportion of frontal lobe onset seizures patients with specific underlying pathologies that are associated with drug resistance such as focal cortical dysplasia (FCD). If our cohort contained a significant number of such structural cases, this could have contributed to the observed statistical association. Four, frontal lobe epilepsy may represent a distinct drug-resistant risk phenotype that emerges through interactions with other features, an aspect that may not have been sufficiently revealed in previous univariate analyses or studies of different populations.

Timely management of epileptic emergencies is a clinical research hotspot. EPS discharge may result in an excessively long-lasting seizures and even cause irreversible neuronal damage.^6^ Clinically, nearly three-quarters of patients with SE present as non-convulsive SE.^38^ In 2015, the ILAE defined the time point for focal status epilepticus with impaired awareness as 10 min.^6^ Among the 470 FIC patients in the included cohort, we first determined that 3 min was the optimal prognostic threshold for DRE through ROC curve. Despite the low Youden index and sensitivity of this cutoff point, the pFIC patient group had significantly more DRE cases. Furthermore, this variable exhibited no collinearity effects, and the predictive model demonstrated good fit. Notably, our study was conducted prior to the release of the 2024 international consensus, which defined prolonged focal seizures as lasting >5 min but not meeting the criteria for status epilepticus.^5^ Despite a minor difference in the time threshold, both our definition and the latest consensus support the key clinical concept that early intervention prior to the traditional time point for SE may be warranted. Our findings therefore provide real-world evidence that aligns with the direction of the latest expert recommendations, further strengthening the clinical relevance and validity of our study. Within the complex spectrum of seizure disorders, EPS occupy a critical intermediate zone; however, their pathophysiological characteristics and clinical outcomes remain understudied. At present, key scientific questions about the neurobiological mechanisms, aetiological associations, and long-term neurocognitive impacts of EPS still need to be clarified. Future research should focus on prioritize establishing standardized diagnostic protocols, developing precise quantification tools, and leveraging multicenter collaborations to establish large-scale clinical cohorts, aiming to provide evidence-based basis for individualized treatment.

The main strength of our study is the inclusion of a large sample of individuals across a wide age range. We accurately distinguished types of epileptic seizures, which make it more representative of epilepsy patients. Secondly, we recorded the duration of each epileptic seizure based on EEG, which greatly reduced the recall bias that relies on the patient and their family's memory of seizures.

Our study has some limitations. First, as this study is retrospective, there is a risk of inaccurate medical records and selection bias, and the follow-up time for each patient is also inconsistent. Second, previous studies have indicated that the duration of the second seizure in children with two or more seizures is highly correlated with the first, suggesting that EPS may occur consistently in certain patients.^39^ However, there may still be cases where the duration of past seizures does not coincide with the seizures duration when performing VEEG monitoring. Since we recorded the longest duration, the number of patients in the pFIC group may be relatively larger. Third, a potential limitation of our study is the reliance on scalp EEG for determining seizure onset. In ∼25% of patients, a measurable delay was observed between clinical onset and scalp ictal discharge, which is likely attributable to volume conduction and spatial filtering effects.^40^ This latency may have resulted in an underestimation of true seizure duration. Fourth, it is regrettable that this study failed to effectively distinguish between patients with EPS and SE, nor was it able to compare the prognostic outcomes of these two groups of patients. Fifth, the research model lacks an external validation, and the universality and stability of the results require verification through multicenter data. Sixth, given our centre's protocol of performing brain MRI primarily for diagnostically uncertain or drug-resistant cases, the higher aetiology identification rate in the DRE group is likely influenced by an ascertainment bias inherent to this retrospective study. Seventh, this study has deficiencies in the collection and analysis of imaging data, failing to fully integrate imaging features for in-depth analysis and quantitative research on the aetiological diagnosis, lesion localization, and prognostic evaluation of epilepsy. This makes it difficult to comprehensively reveal the pathogenesis and pathophysiological process of epilepsy from the imaging perspective.

## Conclusion

In summary, for patients with focal epilepsy experiencing FIC, a longest FIC duration ≥3 min was identified as a significant predictor associated with DRE risk, although its standalone predictive value was modest. It serves as a key component in our integrative prediction model. PFIC, frontal lobe origin, known aetiology, multiple seizure types and subclinical seizures were risk factors for DRE. While previous studies have focused on SE, we have explored the relationship between seizure duration and drug responsiveness, which can aid in early intervention and treatment.

## Supplementary Material

fcag114_Supplementary_Data

## Data Availability

The data supporting the findings of this study are available in the Supplementary material. Raw data can be obtained from the corresponding author upon reasonable request.
