# Supplementary material for: Prolonged focal impaired consciousness seizures: a biomarker of drug resistance in patients with epilepsy
Source: Brain Commun. 2026 Apr 2;8(2):fcag114. doi: 10.1093/braincomms/fcag114 (PMC13069483; doi:10.1093/braincomms/fcag114)
Supplement: fcag114_Supplementary_Data [file fcag114_supplementary_data.docx]

**Supplementary material**

**Supplementary Figure 1: Receiver operating characteristic analysis of longest FIC duration for predicting DRE.**


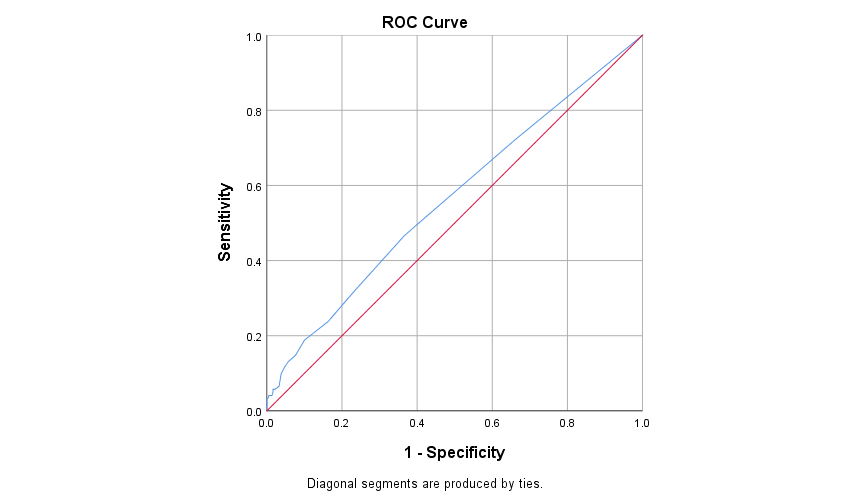


This ROC curve was generated from a single-center cohort of patients with FIC (N = 490). The AUC for the longest FIC duration was 0.571 (95%CI: 0.511-0.631). Using the maximum Youden’s index, the optimal cutoff value was identified as 3 minutes, corresponding to a Youden’s index of 0.111, with a sensitivity of 47.5% and a specificity of 63.6%.

**Supplementary Figure 2: Receiver operating characteristic analysis of prediction model for DRE.**


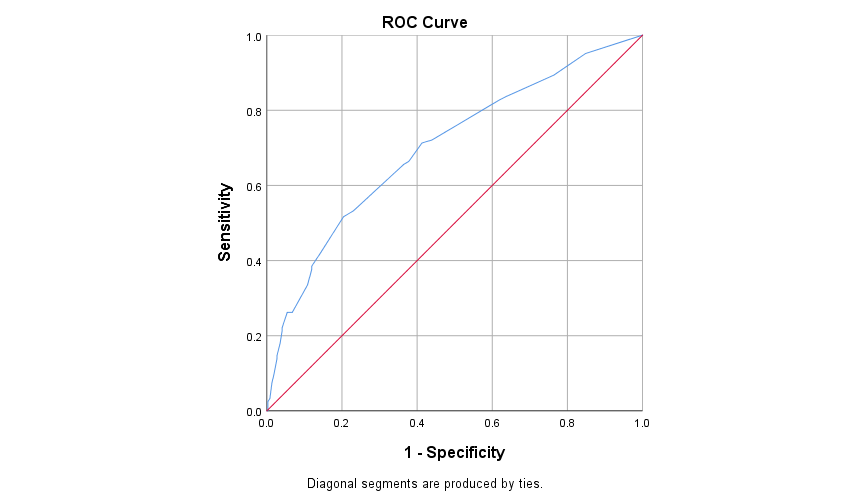


This ROC curve was generated from a single-center cohort of patients with FIC (N = 490). The DRE prediction model incorporated five predictors: pFIC, frontal lobe origin, known etiology, multiple seizure types, and subclinical seizures. The model achieved an AUC of 0.700 (95% CI: 0.645-0.756). The grey diagonal line indicates no discriminative ability (AUC=0.5). These results suggest moderate predictive performance of the model in discriminating DRE.

**Supplementary Table 1: Performance of the longest FIC duration at each possible cut point**

| Longest FIC duration, minute | Sensitivity, % | Specificity, % | Youden index |
| --- | --- | --- | --- |
| ≥2 | 73.0 | 34.2 | 0.072 |
| ≥3 | 47.5 | 63.6 | 0.111 |
| ≥4 | 32.8 | 76.9 | 0.097 |

Abbreviations: Focal impaired consciousness seizures = FIC.

**Supplementary Table 2: Results of the multivariate logistic regression analysis.**

| Variables | Regression coefficient | OR | 95% CI | *P* values |
| --- | --- | --- | --- | --- |
| pFIC | 0.467 | 1.596 | 1.030-2.473 | 0.036* |
| Presence of subclinical seizures | 0.856 | 2.353 | 1.329-4.166 | 0.003* |
| Multiple seizure type | 1.017 | 2.764 | 1.546-4.943 | 0.001* |
| Frontal seizure onset | 0.632 | 1.881 | 1.203-2.940 | 0.006* |
| Known etiology of epilepsy | 0.981 | 2.667 | 1.030-2.473 | 0.000* |

Abbreviations: Prolonged focal impaired consciousness seizures = pFIC.
